# Knocking down Insulin Receptor in Pancreatic Beta Cell lines with Lentiviral-Small Hairpin RNA Reduces Glucose-Stimulated Insulin Secretion via Decreasing the Gene Expression of Insulin, GLUT2 and Pdx1

**DOI:** 10.3390/ijms19040985

**Published:** 2018-03-26

**Authors:** Jie Wang, Wenyi Gu, Chen Chen

**Affiliations:** 1 School of Biomedical Sciences The University of Queensland, Brisbane 4072, Australia; jiewang1986@yahoo.com; 2 Australian Institute for Bioengineering and Nanotechnology, the University of Queensland, Brisbane 4072, Australia

**Keywords:** Type 2 diabetes, RNA interference, shRNA, pancreatic beta cells, insulin resistance, pancreatic beta cell dysfunction, insulin receptor, insulin secretion, glucose uptake

## Abstract

Type 2 diabetes (T2D) is a metabolic disorder characterized by beta cell dysfunction and insulin resistance in fat, muscle and liver cells. Recent studies have shown that the development of insulin resistance in pancreatic beta cell lines may contribute to beta cell dysfunction in T2D. However, there still is a lack of detailed investigations regarding the mechanisms by which insulin deficiency may contribute in diabetes. In this study, we firstly established a stable insulin receptor knockdown cell line in pancreatic beta cells INS-1 (InsRβKD cells) using anti InsRβ small hairpin RNA (InsRβ-shRNA) encoded by lentiviral vectors. The resultant InsRβKD cells demonstrated a significantly reduced expression of InsRβ as determined by real-time PCR and Western blotting analyses. Upon removing glucose from the medium, these cells exhibited a significant decrease in insulin gene expression and protein secretion in response to 20 mM glucose stimulation. In accordance with this insulin reduction, the glucose uptake efficiency as indicated by a ^3^[H]-2-deoxy-d-glucose assay also decreased. Furthermore, InsRβKD cells showed a dramatic decrease in glucose transporter 2 (GLUT2, encoded by SLC2A2) and pancreatic duodenal homeobox (Pdx1) mRNA expression compared to the controls. These data collectively suggest that pancreatic beta cell insulin resistance contributes to the development of beta cell dysfunction by impairing pancreatic beta cell glucose sensation through the Pdx1- GLUT2 pathway. InsRβKD cells provide a good model to further investigate the mechanism of β-cell dysfunction in T2D.

## 1. Introduction

Diabetes is one of the fastest growing chronic diseases worldwide [1]. Type 2 diabetes (T2D) accounts for over 90% with more of the pre-diabetes population exhibiting an elevation of fasting glucose and/or impaired glucose tolerance [2]. Intermediate hyperglycemia can result from deficient insulin secretion and insulin resistance [3]. Impaired response to insulin action in fat, muscle and liver cells induces a breakdown of fat, a failure of glycogenesis, and excess insulin secretion at early stages of diabetes [4,5]. Long-term compensating insulin secretion to insulin resistance may eventually lead to pancreatic beta cell failure [6]. 

Previous studies have suggested that neither peripheral tissue insulin resistance nor glucolipotoxicity fully explain the onset of beta cell dysfunction showing a delayed and insufficient insulin section to hyperglycemia in T2D. The pancreatic beta cell insulin receptor knockout (βIRKO) mice [7] lose insulin secretion in response to glucose, suggesting insulin receptors in beta cells may play a role in beta cell dysfunction. However, deletion (knock out) of InsR expression results in a complete loss of insulin action through life, which is different from progressive impaired insulin action on beta cells. Other studies using a small interfering RNA (siRNA) to knockdown insulin receptor (IRKD) in MIN6 cells described a significant decrease in glucose-stimulated insulin secretion (GSIS) and a reduced cell proliferation [8,9]. The siRNA-induced gene silencing yielded only a short-term suppression with a rapid recovery. It is thus necessary to set up a long-term InsR knockdown model in vitro to investigate the molecular mechanism underlying pancreatic beta cell dysfunction. 

Insulin signalling pathway regulates glucose transport and metabolism. The tyrosin kinase domain of InsR β subunit plays a major role in InsR signal transduction [10,11], thus silencing β subunit of InsR gene in pancreatic beta cells is appropriate to clarify the contribution of pancreatic beta cell insulin resistance. The transport of glucose is predominantly conducted by GLUT2 in pancreatic beta cells. GLUT2 allows a parallel rise of pancreatic beta cell insulin secretion in response to an increase in blood glucose concentration [12,13,14,15]. The loss of GLUT2 expression was found in diabetic db/db mice pancreatic beta cells, which could be restored by transplanting islets from normal mice to diabetic mice [16]. GLUT2-null mice displayed a loss of first (rapid) phase GSIS, which could be rescued by re-expression of GLUT2 in pancreatic beta cells [17].

Pdx1, a major regulator of GLUT2 expression, can bind to the GLUT2 transcription promoter [18,19]. Pdx1 expression is restricted to the β and δ pancreatic cells and confers the downstream expression of pancreatic beta-cell-specific genes, such as insulin, islet amyloid polypeptide, and GLUT2. The binding of Pdx1 and Pdx1-recognized TAAT motif enhances the transcription of the GLUT2 gene [18,19]. Reduced Pdx1 expression results in a decline in GLUT2 expression [18,20,21]. In this study, we firstly set up a stable InsR β knock-down beta cell line and then demonstrated a decreased GSIS in the cells through the reduction of GLUT2 and Pdx1 in the cells. 

This InsRβKD cell line thus provides a good model to further investigate the mechanism of β-cell dysfunction in T2D.

## 2. Results

### 2.1. Cloning shRNAs into pLL3.7 Plasmid and Lentiviral Production 

**Figure 1:**
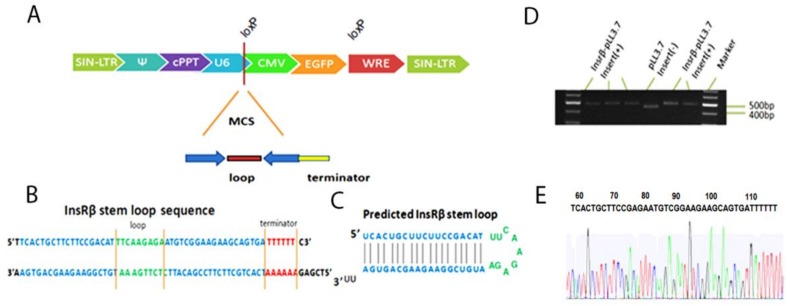
Lentiviral vector and predicted InsRβ shRNAs. (**A**) The linear structure of pLL3.7; the shRNA expression cassette is under the control of U6 promoter and eGFP gene is under the CMV promoter. (**B**) The annealed shRNA expression cassette. (**C**) The finished stem-loop structure of a representative shRNA. (**D**) Agarose gel electrophoresis shows a 500 bp band cut from a positive InsRβ-pLL3.7 while band cut from pLL3.7 was 450 bp. (**E**) Sequencing results confirms the inserted shRNA-3 sequence is correct.

The backbone of lentiviral transfer plasmid pLL3.7 and the cloning sites of Hap I and Xho I are shown in Figure 1A. The sequence and expected stem-loop structure of InsR shRNA were shown in Figure 1B,C. The cloning results were confirmed by both agarose gel electrophoresis (Figure 1D) and sequencing (Figure 1E). The DNA fragment cut from the pLL3.7 vector with no insert gave a band of 450 bp, while the digest containing the InsRβ shRNA gave a slight bigger band of 500 bp (Figure 1D). The sequencing results confirmed the insert of shRNA-3 was correct (Figure 1E). Lentiviruses carrying InsRβ shRNAs and control shRNA 7-14 were produced by co-transfection of 293T cells with transferring plasmid and packaging plasmids as previously reported [22].

### 2.2. Establishment of Stable Insulin Receptor Knocking down Cell Line

**Figure 2:**
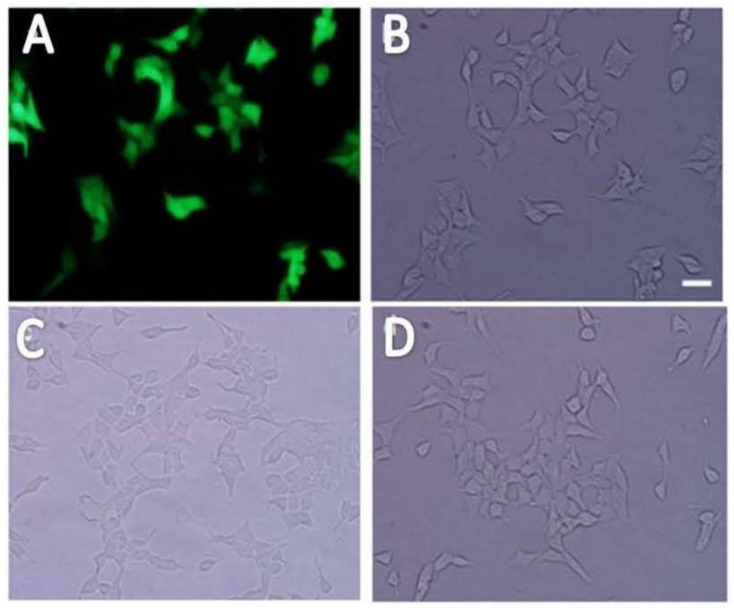
The morphology of transduced INS-1 cells. The transduced INS-1 cells showed strong green fluorescence (**A**) under a fluorescent microscope. The same view of the cells under white light field (**B**) show the cell morphology of transduced cells, indicating most of the cells were GFP-positive. From the view of morphology under the light microscope, the control un-transduced INS-1 cells (**C**) and InsRβKD INS-1 cells (**D**) were no much differences. The scale bar is 50 μm and all images share the same scale.

**Figure 3:**
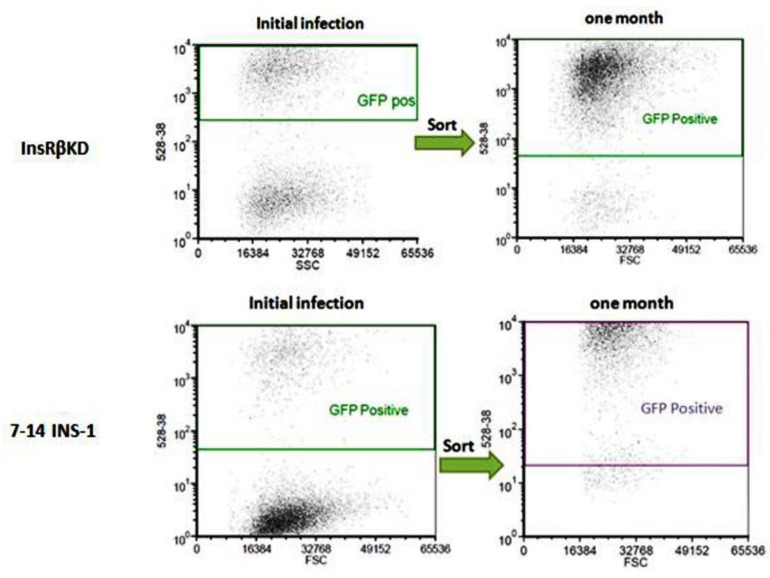
The stability of transduced INS-1 cells. After initial transductions of INS-1 cells with lentiviruses, GFP positive cells were sorted by FACS (**left panels**). After one-month culture of the positive cells, the InsRβKD and LV-7-14-INS-1 cells show high percentages of GFP-positive (**right panels**), suggesting the transduction is stable.

INS-1 cells were transduced with LV-InsRβ shRNAs and LV-7-14 shRNA and examined under a fluorescence microscope (lentiviral vector carries eGFP gene). Under UV light, successfully transduced INS-1 cells showed green fluorescence (Figure 2). Compared to control INS-1 cells, the transduced INS-1 cells maintained the same morphological characteristics (Figure 2). After proliferation of cells to more than 80% confluence, GFP-positive cells (around 50–60%) were sorted by FACS, and the sorted cells were kept in culture for one month to monitor the decline of lentiviral vector. After one month and about eight passages, the sorted cells were still highly GFP-positive (Figure 3), which suggested that transduced INS-1 cells were genetically stable and can be used in the following experiments. 

### 2.3. Confirmation of Gene Silence 

**Figure 4:**
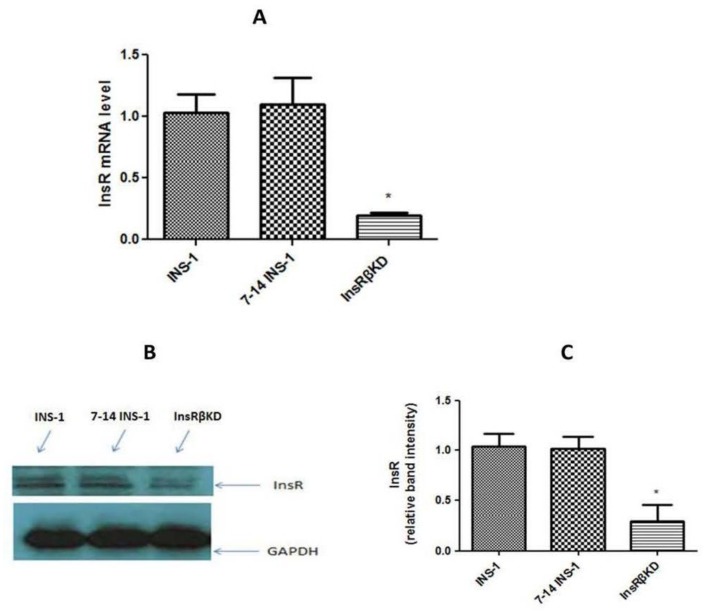
InsR expression in transduced cells. (**A**) qPCR result of measuring InsR mRNA levels in the 3 cell lines. The quantity level was firstly normalized to that of endogenous control glyceraldehyde 3-phosphate dehydrogenase (GAPDH) and then expressed relative to that in INS-1 cells. (**B**) A representative Western blot analysis of InsR protein levels in INS-1, 7-14 INS-1, and InsRβKD cells. GAPDH protein was similarly analysed as the loading control. (**C**) The densitometry analysis of band intensities of InsR relative to these of GAPDH. * *p* < 0.05, *n* = 3.

The InsR expression was firstly analysed by qPCR and Western blot in stable transduced cell lines. Data from qPCR showed a significant reduction (~80%) of InsR mRNA expression in InsRβKD cells (Figure 4A), compared to LV-7-14 transduced and non-transduced INS-1 cells. No significant decrease of InsR mRNA expression was found between the controls (Figure 4A). InsRβKD cells also showed a significant reduction in InsR protein level, while no difference was observed between LV-7-14 transduced cells and INS-1 cells (Figure 4B,C). These data suggest that InsR protein levels are consistent with mRNA expression profiles (Figure 4B) and that specific gene silence has been achieved in the stable cell line InsRβKD. As shRNA-3 showed a better knocking down results than other sequences, InsRβKD cells from this shRNA were used in the following experiments. 

**Figure 5:**
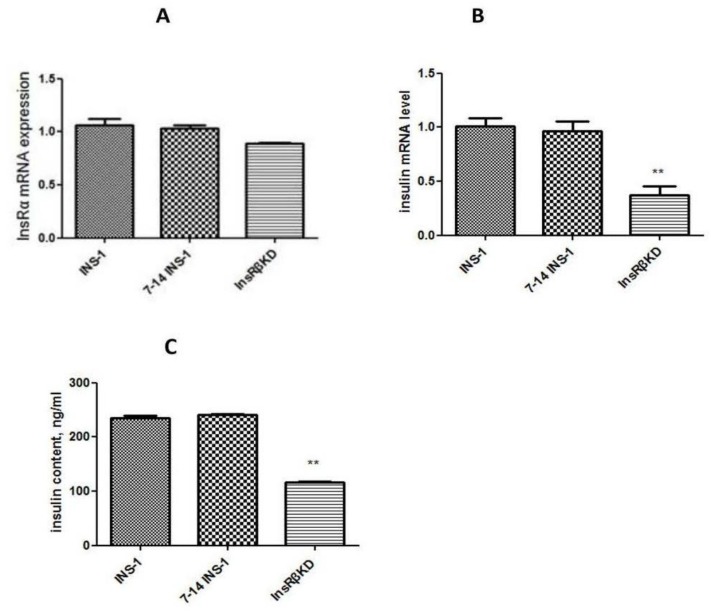
InsRα and insulin mRNA expression, and insulin content in transduced cells. InsRα (**A**) and insulin (**B**) mRNA expressions were measured using qPCR. The mRNA expressions were normalized to that of GAPDH and then to that of INS-1 cells. (**C**) ELISA result of insulin levels in INS-1, 7-14 INS-1, and InsRβKD cells. InsRβKD cells showed a reduction of insulin levels compared to the controls. ** *p* < 0.01, *n* = 3.

To exclude off-target effects of the shRNA, the expression of InsRα was measured by qPCR. Data obtained from qPCR showed a slight (non-significant) reduction (around 10%) of InsRα mRNA expression in InsRβKD cells (Figure 5A). Compared to INS-1 cells and LV-7-14 INS-1 cells, no significant decrease of InsRα mRNA expression was found (Figure 5A).

### 2.4. Reduced Insulin Expression and GSIS in InsRβKD Cells

**Figure 6:**
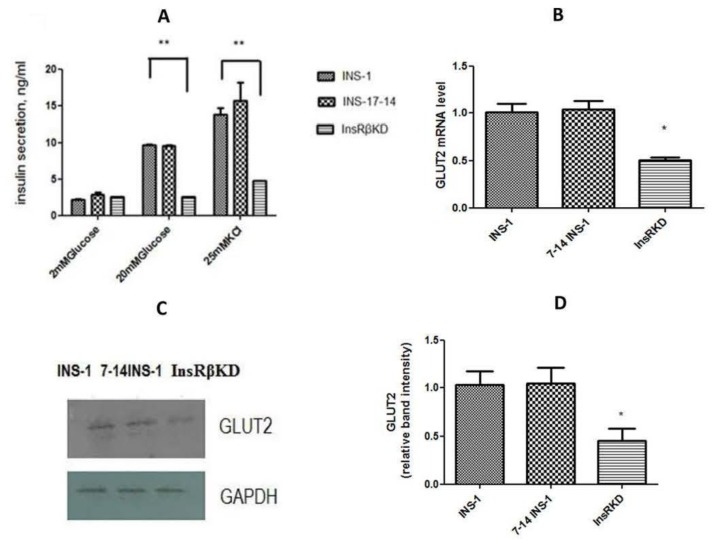
GSIS and GLUT2 expression in transduced cells. (**A**) ELISA results of insulin secretion induced by 2 and 20 mM glucose and 25 mM KCl in INS-1, 7-14 INS-1, and InsRβKD cells. Compared to controls, InsRβKD cells showed significantly reduced insulin secretion at 20 mM glucose and 25 mM KCl stimulations. (**B**) GLUT2 mRNA expression by qPCR analysis, which was normalized to GAPDH expression and then to that of INS-1 cells. (**C**) A representative result of Western blot analysis for GLUT2 protein expression. (**D**) The densitometry analysis of band intensity of GLUT2 relative to GAPDH. * *p* < 0.05, ** *p* < 0.01, *n* = 3.

To investigate the effect of InsR knock-down on insulin production, insulin mRNA expression, insulin content, and GSIS were assessed in transduced cells. qPCR analysis showed that insulin mRNA expression in InsRβKD cells declined relative to that in control cells (Figure 5B). A corresponding result was obtained from insulin content analysis, which indicated a 50% reduction of insulin content in InsRβKD cells in normal glucose culture conditions (Figure 5C). To assess the GSIS, cells were serum-starved in KRB buffer with 2 mM glucose for 45 min and then treated with different concentrations of glucose or 25 mM KCl. Insulin assay results revealed that all cells showed a dose-dependent increase of GSIS and at their highest levels with 25 mM KCL treatment (Figure 6A). InsRβKD cells released less insulin in response to the stimulation of high concentration glucose at 20 mM glucose or 25 mM KCl (Figure 6A). At 2 mM of glucose, there was no difference observed between InsRβKD cells and the controls (Figure 6A).

### 2.5. Reduced Glucose Influx through GLUT2 and Pdx1 Expression in InsRβKD Cells

**Figure 7:**
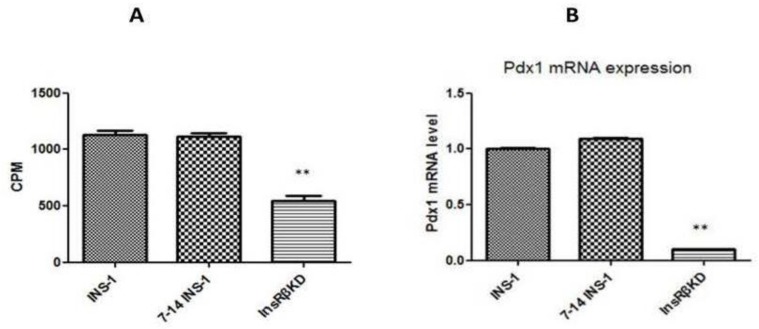
Radioactive 2-deoxyglucose uptake and Pdx1 expression in transduced cells. (**A**) Radioactivity of radioactive 2-deoxyglucose uptake expressed as CPM by a microplate scintillation counter. These results were then expressed relative to those in INS-1 cells. (**B**) The Pdx1 mRNA levels by qPCR analysis that was normalized to those of GAPDH and then to INS-1 cells. ** *p* < 0.01, *n* = 3.

To explore the mechanism underlying the reduced GSIS in InsRβKD cells, GLUT2 mRNA expression was measured by qPCR. The results showed a decrease of GLUT2 mRNA expression in InsRβKD cells compared to the controls of INS-1 and LV-7-14 INS-1 cells (Figure 6B). Western blot data further confirmed the reduced GLUT2 expression in InsRβKD cells after InsR knock-down (Figure 6C,D). Glucose transport activity was assessed by measuring the radioactivity of ^3^[H]-2-deoxyglucose uptake into the cells. To ensure the measured glucose uptake mediated by GLUT2 translocation from cytosol to membrane, a group of cells were treated with cytochalasin B, an inhibitor of actin filament-dependent GLUT2 translocation. The subtraction of cytochalasin B-treated group counts from cytochalasin B-free group counts yielded the actual radioactivity of ^3^[H]-2-deoxyglucose uptake mediated by GLUT2. Compared to samples harvested from INS-1 cells, samples from InsRβKD cells showed a significant reduction of radioactivity, which reflected a reduction of glucose uptake in InsRβKD cells (Figure 7A). To clarify the contribution of the insulin signalling pathway to the decline in GLUT2 expression and glucose uptake, Pdx1 mRNA expression level was analysed by qPCR. A significant reduction of Pdx1 expression in InsRβKD cells was observed, compared to Pdx1 expression levels in controls (Figure 7B).

## 3. Discussion

In this study, we have established an insulin-resistant pancreatic beta cell model using a lentiviral-mediated RNA interference technique. Gene expression analysis (Figure 4A,B) indicated that the designed shRNA specifically induced a reduction of InsR expression. Moreover, the transduction effect in FACS-purified cells was stable over one month and more than eight passages with GFP as a marker of successful transduction (Figure 3). Furthermore, cell morphology indicated no significant cytotoxic effects (cell death) caused by the lentiviral transduction as previous reports [23,24]. In addition, stable expressions of InsRα and GAPDH were also measured to exclude the off-target effects, while the expression of InsRβ was significantly reduced to around 20% of that in control INS-1 cells (Figure 4 and Figure 5A). This indicates that InsRβ shRNAs are highly specific with little off-target effects. 

This lentiviral shRNA-induced gene silencing technique in pancreatic beta cell lines has the following advantages: (1) lentiviruses can efficiently infect mammalian cells; (2) lentiviruses can integrate into the host cell genome, and hence, provide a stable expression of the shRNA; (3) lentiviruses can be used to express multiple shRNAs simultaneously; and (4) the third generation lentiviruses are safe to use and are not replication-competent viruses [25,26,27,28]. Our study not only established InsRβKD cells as an insulin-resistant pancreatic beta cell model but also introduced lentivirus-mediated shRNA as an efficient way for long-term target gene silencing cells. This kind of LV-InsRβ is expected to be a useful tool for further studies of insulin resistance. 

There have been many in vivo as well as in vitro studies attempting to address the question of whether insulin secreted from pancreatic beta cells regulates its own synthesis and/or secretion with conflicting results [29,30,31,32,33]. In the present study, suppressing the insulin receptor resulted in a reduction in insulin gene expression (Figure 5B). Activation of insulin gene transcription depends on the interactions of multiple nuclear proteins on the insulin gene promoter to build a transcriptional activation complex. Many transcription factors have been found to act upon the insulin promoter region, such as Pdx1, MafA, and Beta2 (Neuro D). Pdx1 not only enhances the formation of insulin transcriptional activation complex by binding to the A3/4 region of the insulin promoter but also activates insulin gene transcription by cooperating with the basic helix-loop-helix (bHLH) proteins (such as Beta 2 and E47) that bind to the E1 site of insulin promoter [34,35,36,37,38]. In the present study, Pdx1 was significantly reduced in InsRβKD cells, suggesting that Pdx1 is involved in the reduced insulin expression in insulin-resistant β-cells. The mechanism by which MafA participates in insulin transcription includes binding to and synergizing with Pdx1, Beta 2 [39,40]. These beta cell specific transcription factors interact with each other to tightly control insulin gene expression in a coordinated and synergistic manner. 

As one of the early symptoms of beta cell failure in T2D, impaired GSIS was found in InsRβKD cells (Figure 6A). The first phase GSIS occurs mainly at the level of exocytosis, which results from an increase in the ATP:ADP ratio, the closure of K_ATP_ channels causing cell membrane depolarization, Ca^2+^ influx and insulin release from readily releasable pool. When T2D progresses, total insulin secretion becomes insufficient leading to significant hyperglycemia. In the present study, the loss of insulin secretion at high concentrations of glucose stimulation indicated a reduced glucose metabolism rate and potential defects in cell membrane electrophysiological properties, in addition to deficient insulin. In the present study, the role of glucose transport was assessed and investigated.

The uptake of glucose into pancreatic beta cell plays a key role in the control of GSIS. Glucose uptake by pancreatic beta cells is mediated by GLUT2 and its deficiency is thought to causing diabetes. Mutational analyses of the coding region of the *GLUT2* gene have been performed without consistent finding of linkage or association with T2D [41,42,43,44,45]. The present study provides the supporting evidence that GLUT2 expression plays a crucial role in the regulation of insulin secretion in the pancreatic beta cell line. The contribution of GLUT2 deficiency in the loss of GSIS was further confirmed by glucose uptake assays designed specifically to measure GLUT2-induced glucose influx (Figure 7A). In insulin-resistant InsRβKD pancreatic beta cell line, GLUT2 deficiency may contribute to pancreatic beta cell failure. 

An element thought to control GLUT2 expression is Pdx1, which has been addressed in the present study. Gene expression assay indicated a dramatic decline of Pdx1 gene expression in InsRβKD cells (Figure 7B), which suggested that Pdx1 may play a role in the down-regulation of GLUT2 in insulin-resistant pancreatic beta cells. Taken together, Pdx1-GLUT2 may be one of the signalling pathways underlying pancreatic beta cell-insulin-resistance-induced beta cell dysfunction. 

To conclude, normal InsR signals in beta cells are essential to maintain normal cell function. If insulin resistance or disturbance of InsR occurs, GSIS-related signalling pathways may be affected and β-cell dysfunction may occur, including disturbances in insulin synthesis and secretion. For disturbance in GSIS, Pdx1-GLUT2 and subsequent glucose uptake are involved. 

## 4. Materials and Methods

### 4.1. Small Hairpin RNA (shRNA) Design, Cloning and Sequencing

We designed 4 shRNAs based on Rattus norvegicus InsR β (InsRβ) mRNA sequence (Gene ID: 24954) following the guidelines reported previously [22,46,47]. The shRNA expression cassettes contained 19 nucleotide (nt) of the target sequence followed by the loop sequence (TTCAAGAGA), reverse complement to the 19 nt, stop codon for U6 promoter, and Xho cloning site:

*-InsRβ-1*: 5′TGATGACAGCAGAAATTGCCTTCAAGAGAGGCAATTTCTGCTGTCATCTTTTTTC;
*-InsRβ-2*: 5′TGAATGACGAGAGACATCTATTCAAGAGATAGATGTCTCTCGTCATTCTTTTTTC;
*-InsRβ-3*: 5′TTCACTGCTTCTTCCGACATTTCAAGAGAATGTCGGAAGAAGCAGTGATTTTTTC;
*-InsRβ-4*: 5′TGTTCGAGGACATGGAGAATTTCAAGAGAATTCTCCATGTCCTCGAACTTTTTTC.

These shRNA expression cassettes and their complementary strands were synthesized commercially (Sigma, Victoria, Australia) and annealed in the annealing buffer following a protocol previously described [47]. Briefly, the resulting double-stranded oligo DNAs were cloned into pLL3.7 plasmids at the Hap I and Xho I of the MCS sites (Figure 1A) through phosphorylation and ligation reactions. To check the insert, the plasmids were digested with XbaI and NotI (NEW ENGLAND, Biolabs^®^, Ipswich, MA, USA) and then assessed by 2% agarose gel electrophoresis for the inserted fragment. The plasmids were also mixed with sequencing primer (*5′CAGCACAAAAGGAAACTCACC)* and sent for sequencing (AGRF, Melbourne, Australia) to further confirm the inserted sequences. Because the plasmid also contains an *eGFP* gene, the transduction of the lentiviral vector can be monitor by fluorescent microscope or flow cytometer.

### 4.2. Cell Lines and Maintenance

INS-1 is a rat pancreatic tumor beta cell line, which retains normal GSIS within approximately 80 passages and possesses the same genotype and tissue markers (e.g., glucose transporters, glucokinase, insulin genes) of its parental tissue [48,49]. They were maintained in RPMI-1640 medium supplemented with 10% heat-inactivated fetal calf serum (FBS), 1 mM sodium pyruvate, 50 μM 2-mercaptoethanol, 10 mM HEPES (all from Sigma-Aldrich^®^, St. Louis, MI, USA), 100 IU/mL penicillin and streptomycin (Life Technologies^®^, Carlsbad, CA, USA) in a 37 °C incubator with 5% CO_2_. INS-1 cells were passaged every 5 days when they are 90% confluent. The 293T cells (gift from Rod Minchin, University of Queensland) were maintained in Dulbecco’s Modified Eagle’s Medium (Life Technologies^®^, Carlsbad, CA, USA) supplemented with 10% FBS, 100 IU/mL penicillin and 100 μg/mL streptomycin in a 37 °C incubator with 5% CO_2_. 

### 4.3. Production of Lentiviruses and Transduction of INS-1 Cells

The transferring plasmid pLL3.7 with inserts and packaging vectors (pVSVG, pRSV-REV, and pRRE) were extracted from DH5α E. coli culture with HiPure Plasmid Maxiprep Kit (Life Technologies^®^, Carlsbad, CA, USA). Twenty μg transferring plasmid and 10 μg of each packaging plasmid were co-transfected in 400 μL 1.25 M CaCl_2_, 1.5 mL H_2_O and 2 mL 2× HEPES buffered saline into 293T cells in T75 flasks. After four hours, the medium was removed and the cells were gentlely washed twice with PBS followed by adding 6 mL fresh medium for continuous culture. After 48 h, the supernatant was harvested and concentrated by centrifugation for 50 min at 3000 rpm at 4 °C using Vivaspin 20 mL tubes (Sartorius, Göttingen, Germany). The concentrated lentiviruses were stored at −80 °C freezer. Viral titers were determined as previously described [50]. We also produced LV-7-14 lentivirus as a negative control, the shRNA 7–14 was a non-effective shRNA and previously used as a negative control [51]. 

For transduction, INS-1 cells were plated in 6-well plates at the density of 5 × 10^5^/well and cultured overnight. Cells were then infected by 1 × 10^7^ lentiviral particles in 500 μL complete RPMI-1640/polybrene (8 μg/mL, Sigma-Aldrich^®^, St. Louis, MI, USA) for 60 min at 37 °C before an additional 0.5 mL complete RPMI-1640/polybrene was added. After this, cells were incubated for another 24 h, followed by the replacement of fresh complete RPMI-1640.

### 4.4. Establishment of InsRβKD Cells

After transduction, the transduced cells were sorted for GFP positive cells using fluorescence-activated cell sorting (FACS). The sorted cells were cultured for a month or about 8 passages. These cells were checked for GFP expression again and used for assessing InsRβ expression. If the cells showed a sustained reduction of InsRβ we considered it was a stable cell line of InsRβKD cells. The LV-7-14 transduced cell line was used as a negative control to reveal the potential off-target effects and viral toxicity on INS-1 cells. 

### 4.5. Quantitative PCR (qPCR)

SYBR Green method was used for qPCR and specific primers were designed to ensure the monitored genes have equal amplification efficiency and the length of amplification products are similar. Data were analyzed by 2^−ΔΔCt^ method as previously described [52,53]. For rat actin, GCAAATGCTTCTAGGCGGAC and AAGAAAGGGTGTAAAACGCAGC; for rat insulin Rα, AAAGTTTGCCCAACCATCTG and GTGAAGGTCTTGGCAGAAGC; for rat insulin Rβ, ATGGGACCACTGTACGCTTC and ACCACGTGATGACAGGTGAA; for PDX-1: AAACGCCACACACAAGGAGAA and AGACCTGGCGGTTCACATG; and for GLUT2: CAGCTGTCTCTGTGCTGCTTGT and GCCGTCATGCTCACATAACTCA. 

### 4.6. Western Blotting Analysis

Total protein was extracted from cell culture and InRβ and GLUT2 proteins were quantified by Western blotting assay according to standard protocols. Briefly, cells were harvested by scrapping and lysed in Radio-immunoprecipitation assay (RIPA) buffer. The cell lysate was centrifuged for 5 min at full speed at 4 °C and the supernatant was transferred and stored at −80 °C for further analysis. Protein concentrations were determined using BCA assay kit (Thermo SCIENTIFIC, Waltham, MA, USA) according to manufacturer’ s instructions. Total protein was separated by electrophoresis through 10% resolving gel and transferred to nitrocellulose membrane followed by primary and secondary antibody incubations. Immunodetection was performed according to ECL protocols instructed by the manufacturer. 

### 4.7. Insulin Assay

In 24-well plates, 2 × 10^5^ cells per wells were incubated for 3 days at 37 °C. Cells were washed twice with Krebs Ringer bicarbonate Buffer (KRB) containing 0.5% BSA (Sigma-Aldrich^®^, St. Louis, MI, USA). Cells were then pre-incubated in KRB/0.5% BSA for 45 min, followed by incubation in KRB/0.5% BSA with 2 mM glucose, 20 mM glucose, and 25 mM KCl for 30 min at 37 °C. The supernatant was collected and stored at −80 °C for insulin secretion analysis. In order to determine the remaining cellular insulin content, the cells were washed twice with ice-cold PBS and extracted with 1 mL acidified ethanol containing 75% (*v*/*v*) ethanol plus 15 mM HCl overnight at 4 °C [8,48]. All samples were analysed using the Rat/Mouse Insulin ELISA kit (Merck Millipore North Ride, NSW, Australia) according to the manufacturer’s instructions. 

### 4.8. Glucose Uptake Assay

Different to glucose, ^3^[H]-2-deoxy-D-glucose has the 2-hydroxyl group replaced by hydrogen thus cannot be further metabolized by phosphoglucose isomerase and will accumulate in cells. The accumulation makes equilibration and isotope efflux less rapid than in the case with the 3-o-methylglucose, which is more convenient to be monitored for glucose influx [54,55,56]. To examine the GLUT2 mediated glucose influx, the cells are treated with the GLUT2 inhibitor, cytochalasin B, and the data collected from this group were set as the background [54,57]. In 24-well plates, 5 × 10^5^ cells per wells were seeded and cultured overnight at 37 °C before being used in the assay. To start, cells were washed twice with KRB/0.5% BSA and pre-incubated in KRB/0.5% BSA for 2 h. Then cytochalasin B (Sigma-Aldrich^®^, St. Louis, MI, USA) was added into the wells designed for background signal counting at a final concentration of 12.5 mM. The plate was then kept in the incubator for 1 min. To initiate glucose uptake, ^3^[H]-2-deoxy-D-glucose (gift from A/Prof. Whitehead, Mater Medical Research Institute, Australia) was added to each well and the plate was incubated for 15 min at 37 °C. The plate was placed on ice to terminate the glucose transport and the buffer in wells was aspirated. The wells were washed four times with ice-cold PBS and dried on ice. To determine the radioactivity of cellular content, the cells were lysed by 1% Triton X-100 on a shaker for 15 min. The cell lysate was collected and combined with scintillant (Ultima Gold^TM^ Cocktails, PerkinElmer^®^, Schwerzenbach, Switzerland) for radioactivity counting using a microplate scintillation counter [54,55]. 

### 4.9. Data Analysis

Data were analysed and shown as mean ± S.E.M. Statistic analysis was conducted using one-way ANOVA. *p* value < 0.05 was considered significance.
